# Ecological Exploration of Knowledge and Attitudes Towards Tobacco and E-Cigarettes Among Primary School Children, Teachers, and Parents in Wales: A Qualitative Study

**DOI:** 10.1177/1179173X20938770

**Published:** 2020-08-12

**Authors:** Rachel Brown, Jordan Van Godwin, Lauren Copeland, Britt Hallingberg, Lianna Angel, Sarah MacDonald, Jeremy Segrott, Graham Moore

**Affiliations:** 1 Centre for Development, Evaluation, Complexity and Implementation in Public Health Improvement (DECIPHer), School of Social Sciences, Cardiff University, Cardiff, UK; 2 Cardiff School of Sport and Health Sciences, Cardiff Metropolitan University, Cardiff, UK; 3 Centre for Trials Research, School of Medicine, Cardiff University, Cardiff, UK; 4 Spectrum Consortium, London, UK

**Keywords:** Children, e-cigarettes, tobacco, schools, parents, qualitative

## Abstract

Experimentation with e-cigarettes has grown rapidly among UK adolescents. To date, this topic has been primarily researched in secondary schools, with less understanding of development of attitudes and behaviours at an earlier age. This research reports qualitative data from interviews with pupils, parents, and teachers at 4 case study schools in Wales (N = 42). It draws on Bronfenbrenner’s Ecological Systems Theory to consider how the intersection of systems surrounding primary school-age children and their interaction with these systems, shape knowledge, and attitudes towards e-cigarettes and tobacco. Findings indicate that consistent messaging on smoking from school and family was reflected in strong disapproval among pupils and clear understanding of harms. This was less evident for e-cigarettes, where messages were mixed and inconsistent between home and school, with concerns over what to tell children about e-cigarettes in light of mixed messages and absence of official guidance. Implications of findings for policy and teaching are discussed.

## Introduction

E-cigarette use has risen globally among adults,^1^ including in the United Kingdom where there are an estimated 3.6 million adult users compared with 700 000 in 2010.^2^ To date, growth has been predominantly among current or ex-smokers, and underage use remains low, with 1.7% of 11- to 18-year-olds reporting weekly use.^3^ While experimentation with e-cigarettes has increased among young people, tobacco smoking rates have continued to fall.^4^

Concerns remain over e-cigarettes as a driver for re-normalisation of tobacco^5,6^ with the renormalisation hypothesis evident in policy, including the regulation of e-cigarettes via the EU Tobacco Products Directive 2016 (TPD). However, the argument that e-cigarettes contradict the tobacco denormalisation agenda is countered with suggestions that e-cigarettes could instead further denormalise smoking by acting as a social display of anti-smoking behaviour.^7^ Recent research suggests continuing decline in the acceptability of smoking among young people, providing limited evidence for smoking renormalisation during this period of rapid growth of e-cigarettes.^8^

While there is extensive research on teenage e-cigarette and tobacco use, including the role of family behaviour and peer norms in the uptake of adolescent smoking,^9^ few studies to date have considered the views of primary schoolchildren (below age 12). Understanding the formation of perceptions of tobacco and e-cigarettes in this population, and the role of the school and family context, can inform future policy and intervention approaches. Ecological Systems Theory^10^ provides a tool to frame such an exploration. This considers child development within interdependent and multi-level systems incorporating and surrounding a child^11^ with behaviours influenced by individual factors and interaction within multiple layers of environmental influence.^12^ These layers include the microsystem, referring to structures within the child’s immediate sphere of interaction, including family, school, and peers; meso-system, where system interactions impact the child without necessarily involving them directly, eg, parent-school communication;^13^ exo-system, where neighbourhood and community factors influence development of the child with or without their active participation, eg, through availability of, and exposure, to tobacco and e-cigarettes; and macro-system, relating to wider cultural values and practices. These structures have a bi-directional, interactive relationship with the child, which this framework can aid in exploring.

At the level of family influence, evidence suggests that trying e-cigarettes is more common among children whose parents use either e-cigarettes or tobacco,^14^ raising concerns that the observation of a ‘smoking-like’ act may normalise smoking.^15^ The development of positive attitudes towards e-cigarette use by family may act to stimulate experimentation among youth^16^ and increase risks of nicotine exposure through ready availability of the product.^17^ Alternatively, e-cigarettes may offer parents a means of limiting children’s exposure to tobacco smoking through use in places where children are present, such as in the home or in a car.^18^ E-cigarette use by family may further act to normalise quitting tobacco use, potentially reducing children’s perception of smoking as a socially acceptable and normative behaviour. Family are an important part of the formation of knowledge and attitudes; however, as a relatively new issue, families may be unsure how to discuss e-cigarette use with children,^19^ hindered by conflicting reporting of risk.^20^ Understanding use of, and communication about, e-cigarettes within families can provide insights into these issues and increase understanding of the early development of knowledge and attitudes.

As well as family, schools are significant and are already recognised as a key influence of children’s normative perceptions of tobacco.^21,22^ While some studies have considered school influence on e-cigarette perceptions,^23,24^ evidence to date is limited, particularly for younger pupils. One of the few available studies, conducted in Wales with primary school populations, identified significant variations in pupil knowledge of the function and potential risks of e-cigarettes, which varied by pupil age with older pupils being more informed, and also by whether pupils knew an e-cigarette user or not. The same study also suggested strong disapproval of smoking or vaping by young people, but greater levels of approval of both behaviours when done by adults.^25^ UK schools provide education on smoking harms from a young age through programmes such as SchoolBeat in Wales (https://schoolbeat.cymru/); however, e-cigarettes are not featured in current recommendations and how or whether their use has been incorporated into teaching and school policies and practices is unclear.

In this study, the analysis as a whole is located within a macro-systemic context in which smoking has declined and has become increasingly denormalised through increasingly restrictive regulation, while e-cigarettes have become increasingly prevalent. While we acknowledge the importance of exo-systemic influences, such as availability and community norms,^26,27^ our primary focus is on the micro-systems of school and family, and their meso-systemic interactions. We nevertheless emphasise where interviewees describe these as nested within community-level characteristics, such as local smoking behaviours.

This study builds on existing research by exploring the impact of e-cigarette emergence on children’s understanding of – and exposure to – tobacco and e-cigarette use and by incorporating the perspectives of both parents and teachers as well as pupil data. This article presents qualitative data to consider how the intersection of ecological systems surrounding primary school-age children and their own interaction with these systems results in knowledge and attitude towards e-cigarettes and tobacco.

## Methods

This research adopts a Critical Realist case study approach, which incorporates the subjective reality of participants while acknowledging and identifying the social structures and systems in which behaviour occurs and which act as both enablers of, and constraints to, social practices.^28^ The aim of Critical Realist research is to identify the interactions and components of the research setting that give rise to the observable outcomes present.^28^

### Study design and ethical approval

Data reported here comprised 4 nested qualitative case study schools conducted as part of a larger mixed-method study including a nationally representative survey of 73 schools in Wales, UK (to be reported elsewhere). Ethical approval for the study was obtained from Cardiff University School of Social Sciences Research Ethics Committee.

### Sampling and recruitment – schools

First, 75 primary schools who had taken part in the previous CHETS Wales survey in 2014^29^ were contacted by email, informing them of the opportunity to be involved in survey research. Expressions of interest were invited from all who were contacted to take further part through interviews with pupils and staff, and permit parental recruitment for interview. Of 6 schools who responded, 4 were selected purposively to provide variation in socio-economic status, using region (North and South Wales) and the accepted measure of level of Free School Meals (FSM) eligibility.^30^ Three of those included teach in the English language and one in the medium of Welsh.

### Recruitment – pupils

Information packs were issued to all pupils in Years 4 to 6 (age 8-11 years), containing a letter for them and a version for their parents, with the opportunity for parents to opt their child out of participation. Nine parents returned opt-out forms to exclude their child. Teachers were then invited to select small groups of pupils, representing a range of higher and lower abilities and equal numbers of boys and girls, but with no other specification. In total, 114 pupils took part (Table 1).

**Table 1.** Breakdown of school participants.

School	No. groups	Total	
		Boys	Girls
A	6	14	16
B	6	16	14
C	6	13	17
D	4	12	12
Total	22	55	59

### Recruitment – teachers

Members of the school senior management team were approached for participation in interviews and were provided with information sheets in advance of interview and had the opportunity to ask questions.

### Recruitment – parents

Letters sent home to parents regarding consent for pupil interviews also contained information about parent interviews. This was followed up by teachers at all sites through playground conversations and at parent evenings. The research team attended a parent coffee morning, an afternoon parent group, and made site visits at drop-off and collection time to discuss the project and to collect contact details for later follow-up.

### Pilot testing of data collection tools

To check acceptability and content of consent processes and topic guide for pupils, researchers ran sessions to test materials in one local primary school who had previously helped with similar tasks. Consent was obtained from head teacher, parents, and pupils. Three sessions were run (one group of pupils in each of years 4-6). During the session, understanding of proposed consent and information sheets was tested and language was refined. The draft topic guide was also explored, specifically the use of draw-write tasks and the language explaining these tasks, as well as questions deemed as potentially sensitive. Research documents were then amended based on pupil feedback. It was identified during pilot testing that some pupils felt anxious talking about parental smoking due to their own knowledge that smoking kills. It was therefore decided to stress that children could talk about smoking within their families if they wanted to but they did not have to do so, meaning we cannot provide a proportion of study participants who had family members who smoked or vaped.

### Group and individual interview schedules

Bespoke study topic guides were developed for pupils, parents, and teachers, but with overlapping elements. All interviews were semi-structured, with topic guides used to steer discussions but not to constrain new and emerging areas. Pupils were asked about knowledge of tobacco and e-cigarette function and harms, perceived levels of use around them, school and family practices, and observations of marketing in the local area. For teachers, discussions included challenges to pupil health and school health-based activities, school actions on smoking and e-cigarettes, and working with families. Parents interviews centred around challenges to pupil health and family health-based activities, awareness of school actions on smoking and e-cigarettes, own attitudes and awareness, and communication and family practices on smoking and e-cigarettes.

### Data collection

Fieldwork was completed between June 2018 and January 2019. While we did not aim for continuation of data collection until theoretical saturation had been reached due to the limited time window for data collection, subsequent analysis and the clear and consistent themes within the data suggest that data of sufficient breadth and quality were obtained. Single-sex group interviews with year 4 to 6 pupils were conducted in 3 of the 4 schools, with the fourth preferring mixed gender groups. Half of pupil groups in one school were conducted in the Welsh language (determined by availability of Welsh-speaking research staff). Groups averaged 6 pupils and lasted around 50 minutes, involving a combination of oral discussion and draw/write exercises designed to both obtain information and stimulate conversation. These methods can aid participation of younger children^31^ and increase relaxation in the research environment.^32^ Groups commenced with reading of the information sheet by the researcher to incorporate any differences in literacy levels, followed by an opportunity for questions. The pupil consent form was then completed as a group task, again read by the researcher with time for questions. Teachers remained through this process to assist where pupils had been identified as having additional needs, but withdrew before data collection, remaining nearby for any issues that arose. Three draw/write tasks were completed over the session, first involving completing a sheet with a picture of a cigarette in the centre. Pupils were asked to draw or write anything they knew about cigarettes, including alternative names, smell, how they are used, and any known effects. As the task progressed, the researcher asked questions about what was on the sheets and also invited participants to explain what they had written/drawn. A similar task was then completed using a picture of two styles of e-cigarette, followed by a later task involving participants drawing or writing any places and people that they observed smoking and vaping. Draw/write tasks were interspersed with questions and discussion. To avoid feelings of discomfort or worry over familial behaviour, pupils were advised throughout that they did not have to name any family members who smoked/vaped if they did not want to.

Parent participants (N = 14, 12 female) were sent information sheets in advance of parent interviews and given the opportunity to ask questions. Ten parents were interviewed individually and 4 in a group (recruited from an existing parent group). Interviews were completed with teachers representing senior management roles (Head and Deputy Head) at the case study schools (N = 6, 4 F, 2 M). Teacher interviews were face to face in 3 schools and by telephone in 1. Signed consent for recording and use of anonymised data was obtained, either on the day for face-to-face interviews or in advance for telephone interviews.

### Analysis

All interviews were audio-recorded and recordings were translated smart verbatim (eliminating pauses and fillers). Resulting transcripts, and drawings completed within groups, were first read for familiarity and initial notes made. Visual materials generated by the drawing tasks were read alongside transcripts for that group to ensure inclusion of any content not covered in discussion. All data were then coded and subject to thematic analysis^33^ to facilitate the structured and exploratory elements of the study. Drawing on Critical Realist approaches, analysis incorporated deductive and inductive elements,^34^ through open reading to engage with participant experiences and understanding^35^ and drawing on existing theory (here Bronfenbrenner’s Ecological Systems Theory). Analysis of transcripts was first carried out by RB with pupil, teacher, and parent transcripts treated as separate data sets, meaning analysis of pupil data was done to completion before teacher data and then parent data. The same process was adopted for each participating group. Transcripts were read openly to generate initial themes, before re-reading with the aim of identifying system-level influences. A sample from each dataset was then second coded by JVG, with coding refined further through co-author discussion. After this initial analysis of each dataset, comparative analysis was carried out across the participant groups to generate further insights and with specific focus on domain interactions drawing on Bronfenbrenner’s Ecological Systems Theory.

Data triangulation was approached with the aim of constructing a narrative incorporating diverse perspectives, including areas of both agreement and contrast to illustrate the complexity of social behaviour and processes. Findings were presented regularly to the study management group for discussion and other areas of importance identified through these discussions.

## Findings

Schools are referred to throughout as A to D, and their key characteristics are summarised below, from public information and from researcher reflections following visits:

*School A* is in an area of low socio-economic status and has a high FSM rate. It has around 200 pupils aged 3 to 11, most of whom go on to the local high school (located close by). Its ethos is as a ‘heart of the community’ school, running a parents group for adult learning, and with some teachers having taught multiple generations.

*School B* is situated in an area of low socio-economic status and is a faith school with a catchment area extending outside the locale, which accounts for it being below the national average for FSM. It has over 300 pupils aged 4 to 11, with high ethnic diversity. It is positioned next to a high school, although pupils go on to a range of secondary schools, reflecting the wider catchment area.

*School C* is an urban school situated on a busy thoroughfare, with over 200 pupils aged 3 to 11. The catchment area extends across both lower and higher socio-economic status areas, and the school has an FSM rate well-below the national average. The school was involved in campaigns on air quality and a ‘no smoking near grounds’ approach, which in practice was difficult to monitor due to the volume of passing foot traffic.

*School D* is in a semi-rural location and has over 200 pupils aged 3 to 11 and an FSM rate around the national average. It has a higher proportion of pupils with additional learning needs than the other sites, with lower levels of attainment at age 7 but above national average levels of attainment at age 11.

Data are presented from interviews with pupils, parents, and teachers and explores influences on the development of pupil awareness of – and attitudes to – tobacco and e-cigarettes.

Pupils are referred to by School code, year of study, and sex (eg, A, 4, F). Parents are referenced by number (order of interviews), sex, and relevant school code (eg, Parent 7, M, B), and teachers are referenced by School code.

### Pupil knowledge of tobacco and e-cigarettes

Pupils most commonly used the terms ‘fags’, ‘tobacco’, and ‘nicotine’ when identifying all the words they knew for a pictured cigarette. Several were unsure of the difference between tobacco and nicotine. Groups often involved lively discussion of terms between pupils, as well as questions to interviewers on these issues. Those who knew smokers within the family reported more awareness of different types of tobacco, including pre-rolled and rolling tobacco cigarettes, and this was observed regardless of pupil age. For a similar task on e-cigarettes, the most commonly cited name was ‘vapes’, with others including ‘pens’ and ‘electric cigarettes’. Several pupils, particularly those who reported not knowing an e-cigarette user themselves, did not recognise the images presented, but knew the word ‘vapes’ when another pupil said it. Knowledge of similarities and differences between e-cigarettes and tobacco varied depending on whether the child reported an e-cigarette user in or close to the family, with those who knew a user often displaying more detailed understanding:

My dad does and my mum does, so I know a lot about vapes. (A, 4, F)

Those who described a vaper in the family appeared more knowledgeable about how devices worked, and more likely to know about variable nicotine content in e-liquids. As with tobacco, pupil knowledge was not observably different by age, with personal relationship to a user appearing to be more important. For those who did not describe knowing any users, this limited penetration of the product into their immediate social systems was accompanied by limited knowledge of – and interest in – e-cigarettes.

### Exo-system contributions: smoking and vaping in the local community

All parents suggested that smoking had noticeably decreased in recent years, although some of those from schools A and B suggested that rates remained high within their local communities. Observation of smoking was most common in town centres, described by most participants as being done by both adults and teenagers, and around pubs, with several children suggesting an association between smoking and alcohol:

Yeah, cos there’s loads of like drunk people smoking. That’s, that’s probably most of the cause. (C, 5, M)

This was relatively similar for e-cigarettes, with a mix of adults and teens observed in public places such as town centres. However, it varied in relation to other locations, with less recollection of adult vaping near pubs.

Pupils reported seeing groups of teens smoking in local areas such as parks, with this often described as intimidating and something to be avoided, reinforcing the perception of smoking as a ‘bad’ behaviour:

I live by a park and in the park, it’s always around the same time at 6 o’clock but these teens and they sort of smoke. It’s not nice. (C, 5, F)

This was not observed as often in such places for e-cigarettes. Two of the 4 schools are in close proximity to a high school, and these pupils were more likely to report teens smoking and vaping in the vicinity of the school, particularly at the start and end of the school day. Among parents, vaping was discussed as the latest trend, representing what smoking would have been ‘in their day’:

I mean myself growing up, smoking was one of them things that you tried, it was disgusting and you stopped or you tried later on, as I did . . . So I think it’s becoming more and more, it’s almost like it’s cool to vape, like it used to be cool to smoke so to speak. (Parent 7,M, B)

There was frequent reflection among parents on how social norms of smoking had changed since their youth, with smoking now largely perceived as anti-social and not cool, particularly by their children and similar-aged peers. Some described the shock experienced by their children on trips abroad where smoking was more prevalent and reflected on how much things had changed in the United Kingdom:

. . . we went to France on holiday last year I think they were quite taken aback, by the publicness of smoking. (Parent 8, F, C)

Teachers also discussed their observations of changes to smoking prevalence and impacts in the classroom. Several stated that children were just less aware of smoking now but also reported that it was more obvious which pupils came from smoking households, often because items such as school books smelled of smoke:

Yeah, there’s the homework book, which I notice when I get it. When you mark them at home, you can tell straight away because the homework book, you know, it smells of cigarette smoke. (C, Teacher)

Many children reported seeing e-cigarette promotional material and advertising in the local area, including for stop smoking services, and also seeing e-cigarette use promoted as a smoking cessation aid:

When I drive down the street to get to school there’s a sign in the shop that says ‘Stop smoking’ and then I thought that’s really good, and then I looked underneath and it says ‘Start vaping instead’. (B, 4, F)

For several pupils who had seem similar advertising, the positioning of vaping as a healthier choice was met with some cynicism and was perceived as a sales approach, therefore differentiated from genuine health messages, for example:

When vaping started, it was shops who were selling them started saying this is amazing and so much better, just so that people could buy them, even though they weren’t actually that good for you. (C, 6, F)

A majority of pupils were aware of a local shop selling e-cigarettes, either in the town centre close to the school or, for School C, on the road directly outside. Although this proliferation of shops was a concern to some teachers and parents, who feared vaping being more appealing to children through increased visibility, it did not appear to be of interest to pupils, and brand name recall, including store names, was very low. Other locations of sale for e-cigarettes and e-liquids included markets, newsagents, and supermarkets, where some pupils contrasted the visibility of products with that of smoking. Reported observation of online advertising for vaping was low but, where it did occur, was almost always through video streaming sites, with pop-up ads encountered during watching of video clips and favoured video bloggers – ‘It (vaping ad) was just on YouTube. It just popped up’ (A, 6, M). It is unclear from available data whether these were age-restricted sites or not.

### Micro-system influences: pupil recall of family conversations about tobacco and e-cigarettes

At all sites, smoking was almost universally met with disapproval, with children suggesting that they disliked seeing it. Many showed sophisticated understanding of how smoking norms had changed over time, reflecting discussion in interviews with parents, with some suggesting that many adults smoked because it was just more common in the past. Children often exchanged stories of conversations with older relatives of how ‘everyone smoked’ when they were young:

My aunty told me, when she was younger she saw kids at her school smoking and she thought it was really cool and all the cool kids did it, so she decided to do it. (C, 4, M)

Most were able to speculate on reasons why people might smoke, including stress, getting addicted, being pressured into it when young and that it was more common in the past, ie, ‘everybody did it’. However, it was also often suggested that they simply could not understand why anyone would do it, specifically because of the known health harms and the cost. This was often accompanied by lack of understanding of why smoking was allowed at all, suggesting awareness of macro-level influences on smoking behaviour:

. . . but what I think the government should start doing is sort of making them illegal . . . they’re really dangerous but lots of people even if they don’t smoke they sometimes die from cigarette smoke if they’re around that person a lot. (C, 4, F)

For e-cigarettes, use was most likely to be understood as a way for adults to stop smoking, particularly where the child had an adult in the family who had tried or succeeded in quitting smoking through vaping:

I spoke to my mum about my nana using the electric one. She said it’s because she stopped the tobacco, but she can’t stop smoking, so that’s why she uses the electric one. (B, 6, M)

Among those who talked about knowing someone who had tried to quit smoking through e-cigarettes, this was broadly considered a positive step, but with the caveat that children would prefer their family member to eventually cease use of e-cigarettes as well. For a small number, where a family member had tried to quit this way but either returned to smoking or been vaping longer term, they were more likely to be disparaging about e-cigarettes and their supposed helpfulness. For example, one boy described his mum and dad trying to switch from tobacco to vaping:

. . . When you’re on that and you try and stop, you just get addicted to this (e-cigarette) instead of that, that’s what I think. (B, 5, M)

All children demonstrated knowledge of smoking, and smoking harms, due to receiving lessons in school; however, those children who were most knowledgeable about both smoking and e-cigarettes were those who had a family member who used one or both. Most children reported being warned about smoking by at least one family member. Where children reported that no conversations had taken place, this was typically among those who also reported no smokers in their circle of family and family friends:

Because I don’t really need to talk about smoking, because there’s no one really in my family (who smokes). (C, 6, F)

For children who had received warnings about the harms of smoking from adults who smoked themselves, this could be seen as confusing or contradictory:

P5: My aunty she smokes and she has a no smoking sign on her car.

P6: That’s weird. Why don’t you just put it in the house?

P2: She would learn, you should sneak a no smoking sign in there, on the wall. (A, 4, M)

Family discussion of smoking was not always adult-led, with several children reporting that they had tried to talk a smoking family member into stopping, often prompted by their lessons in school and driven by newly discovered health risks. This was mostly not met with the desired response, resulting in tension and frustration for the child:

P1: I always try to stop my mother smoking them, but she says ‘Okay I’ll stop today’, but the next day she keeps on doing it.

P3: I’ve talked to my aunty about it because she was smoking in the park, and I told her to stop it, and I told her all the reasons why and she said ‘I don’t care, I’ve been smoking for this long, there’s no point in me stopping now’. (A, 4, F)

Family conversations on e-cigarettes were reported almost exclusively by children who had a user in the family, with the issue not often arising in families with no users. Most of these were led by the child asking the person about the device, most commonly resulting in family members explaining use as for stopping smoking. There was less reporting of children pressing e-cigarette users to give up than for tobacco smokers in the family. Children who lived with an e-cigarette user were often keen to display their knowledge in groups, including informing others of names, how the devices worked, and the range of available flavours:

They’re better for you than normal cigarettes . . . Vapes have got different flavours and in town there’s a shop called XXXXX. And you can make different stuff out of it, out of the smoke. My brother he always makes like circles with it. (A, 4, M)

There were some variations, where children who did not know a user had also initiated conversations with parents and family, prompted by observations of public use or smelling vapour when walking behind an e-cigarette. When such conversations were prompted by the child, they reported that it was generally explained as something adults did to stop smoking.

### Parents’ management of communication on smoking and e-cigarettes

Communication on smoking and e-cigarettes was also discussed with parents. Seven were ex-smokers, with two currently using e-cigarettes to avoid smoking relapse. In discussion of smokers in the extended family (defined for discussion as those who were seen relatively regularly by the children), responses were divided between those who reported feeling like they knew quite a few smokers and those non-smokers who tended towards knowing few smokers in their wider communities, including extended family and friendship group:

No, even our extended families we don’t have actually any smokers . . . I don’t know anyone that we know that smokes really. I am trying to think, friends that they go to. I mean there are friends that they go and stay with, or play with, none of them as far as I know smoke. (Parent 3, F, C)

This delineation into ‘smoking’ or ‘non-smoking’ family identities was also reported in relation to vaping by both parents and children, who tended to either know several users or none/very few.

All parents reported feeling that they had good knowledge of smoking harms, in strong contrast to e-cigarettes where most felt that they did not have a clear understanding. Cited harms for e-cigarettes included mechanical risks, eg, devices ‘blowing up’; addiction to nicotine; and unknown chemicals in liquids. Despite lack of clarity on the evidence of harms, all but one suggested that e-cigarettes were safer than smoking; however, this was always qualified with the opinion that as-yet unknown harms would emerge in the future with further research:

I don’t think people, know the full . . . you know what is going to happen, whether they are going to be [as] dangerous as smoking or not for now. I mean there is a lot of research being done on them, but nobody knows the full details. So it might be 10 years down the line . . . (Parent 1, F, A)

The facts behind e-cigarettes are probably as clear as Brexit (Britain’s withdrawal from the European Union, which was ongoing at the time of interviews). No one knows what the bloody hell’s going on or why it’s happening or what the outcome’s going to be. (Parent 2, M, D)

It was common for parents to report that they had discussed smoking with their child(ren) and had communicated strong anti-smoking attitudes. Of these, several reflected that the discussion had been prompted by the child asking questions, promoted by observation of either a family member, family friend, or someone seen in public smoking. A smaller number (of those non-smoking parents) suggested that they had not yet discussed it as they wanted to shield the children until they were older or they brought it up themselves:

INT: Okay, do you plan on having any other sort of conversations with them about smoking or e-cigarettes . . . ?

I don’t think I would make a point of having a conversation . . . But you know as and when it comes up, yeah I will have those conversations with them. (Parent 8, C, F)

This was more commonly reported among those who defined themselves as ‘non-smoking families’, who felt that their child’s exposure to smoking was so infrequent as to make the conversation redundant or premature.

Of those who had discussed smoking, most also suggested that they had communicated anti-vaping attitudes to their child, but with more variations in the way e-cigarettes were described. Definitions included use as still bad but less bad than smoking, to e-cigarettes being as bad as or the same thing as smoking due to ongoing addiction:

(Parent 11, F) My best friend gave up smoking and she now vapes, she’s done it for three years. She just can’t stop vaping now.

INT: Is she happy to stay with it, or is she looking to stop vaping.

(Parent 11, F) She’s happy to stay with it because she thinks it is not as damaging as cigarettes.

(Parent 13, F) Yeah, and people that do it, in their mind it’s a healthier addiction. (Group interview, parents, A)

Those who described wanting to shield their child from smoking adopted a similar approach to not mentioning e-cigarettes and, again, were more likely to report not being around users in their family or social circle.

### School-level influences on pupil knowledge and perceptions of tobacco and e-cigarettes

All schools delivered education on smoking harms through the SchoolBeat programme and, in three schools, with additional content by teachers through the science curriculum. Standardised content through SchoolBeat meant that teaching on smoking was relatively straightforward, although it was noted in two schools (A, B) that teachers were mindful of avoiding frightening pupils whose parents smoked when discussing health harms:

As a teacher, I hold back a little bit if truth be known, purely because those children whose parents smoke, the children don’t choose that and they straightaway, some children say it can cause cancer, and you don’t want children going home with the weight of the world on their shoulders. (B, Teacher)

A majority of pupils recalled this content on smoking, which constituted the primary source of knowledge on health harms and most were able to list health conditions associated with smoking, even where they were unclear on what those conditions meant (as evidenced by questions to the researchers such as ‘what is heart disease?’). There was significant overlap in the harms described, reflecting the uniform curriculum content in schools and reinforcement of key messages at – and from – home. Most children expressed resulting fears of the impact of secondhand smoke, both as something that may cause illness but also as a potential cause of addiction for those exposed to it. They often described tactics used in public to avoid smoke, including changing direction or holding their breath when walking past a smoker in public:

P1: Well my mum told me if you see somebody smoking, then walk a bit quicker or try and hold your breath.

P2: That’s what I do anyway. In town there’s loads of people and I’m like [breathes in and holds breath], because I don’t want to breathe it in. (C, 4, F)

Teachers discussed other school-led activities on smoking, involving pupils in designing posters and leaflets publicising the ban on smoking on school grounds and outside gates. Most parents were either aware of this and reported having seen the materials, or stated an assumption that both smoking and vaping would be banned around the school site. Both teachers and parents suggested that it was now almost unheard of to see parents smoking or vaping on school grounds, suggesting that this assumption is well-founded, implying a lack of normalisation of smoking and e-cigarettes use.

School action on e-cigarettes was less straightforward, with no inclusion in curriculum content or the SchoolBeat programme at the time of data collection. Teachers were likely to report being unsure of what to teach on e-cigarettes and would need additional resources and guidance to feel confident:

INT: . . . would you bring e-cigarettes into that (school lessons) straight away as well?

P2: Now, yeah, definitely.

INT: And what sort of content do you think around that?

P1: I think we’d have to learn a little bit more about it. I suppose it’s giving teachers a bit more information about the dangers as well . . . because I don’t feel completely knowledgeable. (A, paired teacher)

Teachers’ own understanding of e-cigarettes varied significantly, with several stating that they were unsure of harms but likely to take a cautious approach with pupils in the absence of clear knowledge. This was driven by fears of e-cigarettes being appealing to young people, through attractive flavours and by being seen as less harmful than smoking:

I think they’re branding e-cigarettes in a clever way with the flavours and so on, which possibly could entice young people to try them because they look interesting where cigarettes don’t look interesting anymore. (C, teacher)

Fear of renormalisation of smoking was evident in discussion with teachers, including concern that positivity towards e-cigarettes, as well as exposure to nicotine, may increase likelihood of future smoking among young people. Where use had been discussed in school, it had been initiated by pupils during lessons on smoking, with some suggesting that they were then likely to treat it as equivalent to smoking. This was motivated by wanting to keep it as clear as possible for pupils:

I don’t differentiate between the two because I see them both the same.

INT: So you teach it as being basically all the same?

People haven’t really got the idea that it’s probably causing similar damage or perhaps not as much, I don’t know enough about it myself. (C, teacher)

Pupil reports of this content varied and there was some active discussion in groups on whether e-cigarettes had been taught in lessons, with some suggesting that they remembered this and others disagreeing, indicating an overall lack of clear recall:

PAR1: We learn more about cigarettes in school instead of vaping.

PAR3: I don’t think we’ve ever learned about vaping before?

PAR2: No, we’ve only learned about cigarettes. (C, 5, F)

Teacher aims of clarity of approach also meant that e-cigarettes were included as standard in statutory smoking bans on or near school grounds and had featured in posters and leaflets informing parents of this, with a strong sense that it was safest to treat them the same way. Only one school (A) reported a formal non-smoking policy (which governed behaviour of staff and parents on site), with others suggesting that they had not needed to formalise it and had managed through ‘common-sense’ practice instead. The resulting approaches and actions were described as largely the same regardless of whether contained in a written policy or not.

### Meso-system level interactions between family and school

A relatively large proportion of pupils cited having a family member who smoked, and this tended to be communities where many lived close to extended family and smoking was often reported to be inter-generational. For those who lived with, or were close to a smoker, awareness of harms from school was reinforced at home, with reference frequently made to the graphic on-packet warnings on tobacco packets of family members:

PAR3: Lung cancer.

INT: Yeah, so where have you heard of that then?

PAR3: On the back of the tobacco packet.

PAR2: They (on-pack images) look horrendous, they have swelled up chins, they’ve come out here and they’re proper swelled up. (D, 6, M)

This had prompted bi-directional exchanges, with several children suggesting that school learning and seeing on-pack warnings had prompted them to ask their relative to quit.

Among parents, knowledge of school input on smoking was mixed, with some familiar with lesson content and others not, but with a widespread view that primary schools should teach children about smoking harms. However, two parents, both of whom identified as from ‘non-smoking families’ expressed concern that smoking should not be introduced at too young an age:

I mean the thing is with smoking for me it was keeping it out of the children’s radar for as long as possible. (Parent 7, M, B)

Some suggested that the key time was just before transition to secondary school where new challenges and behaviours would be encountered:

. . . and I know when she (older daughter) went to high school she had a big shock at the number of kids that were smoking . . . Part of me I think just wants to protect them from all of that for as long as possible. But having experienced that shock that she had, I don’t know maybe towards the end of Year 6, maybe just preparing them for that. (Parent 8, F, C)

Most of these parents also suggested that e-cigarettes should be included in lessons and were seemingly happy for schools to treat them as equivalent to smoking so as to provide clearer messages for children:

Yeah I think it should include vaping, because even though it’s not as harmful as smoking . . . I’m sure something will come out in the future about that, but yeah I think that should be encouraged not to be done as well. (Parent 6, F, A)

However, this was not shared by all, with one parent who used an e-cigarette himself suggesting that it would be wrong for schools to suggest equivalence of harm based on current evidence. For him, this contrasted with his conversations with his child on his own reasons for vaping, highlighting a potential difficulty for schools in assessing possible lesson content and balancing evidence with deterrence.

## Discussion

This research considers ecological influences on the development of knowledge and attitudes on tobacco and e-cigarettes among primary school children in Wales. It draws on Bronfenbrenner’s Ecological Systems Theory to frame the analytical process and presentations of findings with specific focus on the most proximal influences to the child, illustrating exo, meso, and micro system influences and the interactions between these levels. Results suggest that, for this age group, the family is of primary importance in the development of knowledge and attitudes towards e-cigarettes, with school input currently more limited. Both school and family were both described as influential in relation to attitudes and knowledge towards tobacco.

While our primary focus was on family and school influences, these interactions were often situated by participants within discussion of their local communities. These exo-system influences were described through higher or lower rates of exposure to tobacco and e-cigarettes in the local area, with proximity to the nearest secondary school a factor in relation to teenage behaviour. Evidence suggests that neighbourhood prevalence is important and, if interacting with higher family approval of smoking may increase likelihood of youth smoking.^36^ Although family approval was low here regardless of smoker status, exposure to more modelling of smoking and e-cigarette use may be influential in increased likelihood of e-cigarette experimentation.^29^

Most children were able to distinguish tobacco from e-cigarettes and were aware of key differences in function, although levels of knowledge were highly variable. In relation to individual development and consistent with previous findings, children with e-cigarette users in the family were more informed on how the products work. However, contrary to previous findings, no notable differences were identified here between pupils by age across years 4 to 6, contrasting with previous findings.^25^

Children who reported a relationship with an e-cigarette user also appeared keen to share this knowledge within groups. Those children who had a close relationship with an e-cigarette user also reported more initiation of conversations with that user and asking more questions when exposed to this use. It is unclear whether this indicates a greater level of active interest in e-cigarettes than among children without a close relationship to a user or a more passive function related to simply being in an environment where they are being used. However, the potential role of information seeking should be explored further, as it may be important in future susceptibility to experimentation. Evidence from US research suggests that information seeking on vaping was predictive of increased chances of use later.^37^ This suggests a potential pathway whereby youth who are more interested and better informed may be more likely to try an e-cigarette later, although longitudinal data are needed to explore this further, including any progression to regular use. Qualitative research with adolescents from the United Kingdom has also highlighted the increased opportunities for children living with e-cigarette users to access vaping equipment and nicotine,^17^ although it is not clear to what extent this translates into future experimentation and use.

In relation to childhood learning on tobacco, micro-system contributions and interactions lead to a significantly clearer message on tobacco than for e-cigarettes. Clarity of evidence on tobacco harms has facilitated the development of school curriculum content which delivers consistent, clear messaging on smoking. Multiple and sustained public health measures, including smoke-free spaces, on-packet warnings and display bans have also ensured that public understanding of smoking harms has increased, evidenced by widespread support for such policy measures.^38^ Here this was seen in the consistent messaging on tobacco communicated by parents to children and in parental perceptions of being well-informed on smoking harms, with no discernible difference in this between those who defined as non-smoking families and those with higher numbers of smokers in their social circle. This clarity operated as reinforcement of health harms between school and home, which may collectively have contributed to strong smoking disapproval among pupils.

This flow of information between system levels was also bi-directional, with children displaying active agency in conversations and frequently taking learning from school back home and into discussions with family about smoking. This included communicating strong disapproval to smoking family members and the desire for them to quit due to fear of health harms. Youth agency as a driver for change has been explored in health research, including in healthy eating,^39^ and through ‘pester power’, suggested as a potential mechanism to encourage adult smokers to quit.^40^ However, this should be approached with caution, as attempts here were generally not well-received and were described as unsuccessful, with the potential for resulting conflict, both within the family and possibly between schools and parents where messages delivered at home and school vary in content.

The consistency of tobacco messaging was not as evident in relation to e-cigarettes, where absence of formal curriculum content, as well as higher levels of public confusion,^41^ meant that teachers and parents reported being unsure what to communicate. At present, there is a lack of evidence of effective school-based programmes on e-cigarettes,^42^ resulting in a paucity of guidance for teachers, who here responded by either not discussing e-cigarettes or by treating smoking and e-cigarettes as equivalent during lessons. This absence of clarity of message was also present within families, with parents likely to report being unclear on vaping harms and less likely to have initiated conversations with their children. This was reflected in both limitations to pupil knowledge compared with smoking and also in less evidence of bi-directional communication between child and micro-system levels, specifically parents and school, with children more likely to acquire e-cigarette knowledge from a user they know than either teachers or non-using parents. In later adolescence, while family function retains importance, peer influence assumes greater significance on attitudes and behaviours for smoking,^43^ suggesting that communication from family at this age may be highly important in future outcomes. Absence of clear messaging on e-cigarettes within families and from schools means inevitable variations in pupil understanding that is more observable than for smoking, where curriculum content is established and harms are well-evidenced. Such gaps in knowledge are important in light of public and media debates which are increasingly polarised and confusing. Although it is understandable that schools are likely to equate e-cigarettes and smoking in both educational content and policy, primarily due to lack of clarity on what else to do, this may contrast with family input for children who are close to e-cigarette users within the family. Several children who knew users reported an interpretation of e-cigarettes use as a positive choice by family members due to associations with quitting smoking, with those family members more likely to differentiate between smoking and e-cigarettes at home.

### Strengths and limitations

This qualitative research generates insights into the views of a broad range of stakeholders and is one of the first to include the views of both teachers and parents alongside that of primary school pupils. It adds to understanding of communication around a relatively new health behaviour across multiple domains both including and around younger children. As with all similar research, it does not facilitate claims of representativeness and, as such, further research is recommended with same age pupils in other areas as well as with wider parent groups to understand whether the identified themes occur elsewhere and the potential implications of this for policy and practice. It is also possible that the group context of pupil interviews impacted pupil disclosures and this should be considered. Furthermore, as this is cross-sectional research it cannot detect change over time and the translation of pupil views into future smoking or vaping behaviour. Longitudinal research is therefore recommended to monitor change over time and the effects of age on domain interactions involving children, schools, and families.

The study benefitted from pilot testing of materials to increase confidence in both consent procedures and pupil understanding of data collection tools. The inclusion of second coding of a sample of transcripts, as well as multiple team discussions, also strengthens the analysis process.

### Study implications

In this research, school communication on e-cigarettes was less consistent and evidence led-than for tobacco, reflecting the absence of standardised teaching content available and also the absence of clear public messaging on e-cigarettes to date. This led schools to simplify their approach to e-cigarettes, either excluding them altogether or equating them with tobacco when communicating with pupils. Should schools continue with an unambiguous position, there is a risk of a credibility gap for those receiving different messages at home. Further research is recommended on the development of age-appropriate teaching content for use in schools, which reflects the current evidence-base on e-cigarettes harms and which can reduce the conflicting messaging observed within this study. Research is also recommended to explore the impact of school communication which stresses e-cigarettes as a product for adult smokers and unsuitable for non-smokers. Analysis of our survey data collected shortly after qualitative interviews and published elsewhere^14^ found that children who reported perceiving that e-cigarettes as cessation aids were less likely to report that they might smoke or vape themselves in the near future. Such messaging, which stressed the relationship of e-cigarettes to smoking, may act to build on the strong disapproval of smoking evident in children at this age while acknowledging current ambiguity about health harms and avoiding the risk of loss of credible content.
